# Functional Analysis of Phosphorylation on *Saccharomyces cerevisiae* Syntaxin 1 Homologues Sso1p and Sso2p

**DOI:** 10.1371/journal.pone.0013323

**Published:** 2010-10-11

**Authors:** Qiang Yuan, Jussi Jäntti

**Affiliations:** Research Program in Cell and Molecular Biology, Institute of Biotechnology, University of Helsinki, Helsinki, Finland; CNRS, France

## Abstract

**Background:**

The *Saccharomyces cerevisiae* syntaxin1 homologues Sso1p and Sso2p perform an essential function in membrane fusion in exocytosis. While deletion of either *SSO1* or *SSO2* causes no obvious phenotype in vegetatively grown cells, deletion of both genes is lethal. In sporulating diploid *S. cerevisiae* cells only Sso1p, but not Sso2p, is needed for membrane fusion during prospore membrane formation. Mass spectrometry and *in vivo* labeling data suggest that serines 23, 24, and 79 in Sso1p and serines 31 and 34 in Sso2p can be phosphorylated *in vivo*. Here we set out to assess the contribution of phosphorylation on Sso protein *in vivo* function.

**Principal Findings:**

Different mutant versions of *SSO1* and *SSO2* were generated to target the phosphorylation sites in Sso1p and Sso2p. Basal or overexpression of phospho-mimicking or putative non-phosphorylated Sso1p or Sso2p mutants resulted in no obvious growth phenotype. However, S79A and S79E mutations caused a mild defect in the ability of Sso1p to complement the temperature-sensitive growth phenotype of *sso2-1 sso1Δ* cells. Combination of all mutations did not additionally compromise Sso1p *in vivo* function. When compared to the wild type *SSO1* and *SSO2*, the phosphoamino acid mutants displayed similar genetic interactions with late acting *sec* mutants. Furthermore, diploid cells expressing only the mutant versions of Sso1p had no detectable sporulation defects. In addition to sporulation, also pseudohyphal and invasive growth modes are regulated by the availability of nutrients. In contrast to sporulating diploid cells, deletion of *SSO1* or *SSO2*, or expression of the phospho-mutant versions of *SSO1* or *SSO2* as the sole copies of *SSO* genes caused no defects in haploid or diploid pseudohyphal and invasive growth.

**Conclusions:**

The identified phosphorylation sites do not significantly contribute to the *in vivo* functionality of Sso1p and Sso2p in *S. cerevisiae*.

## Introduction

Eukaryotic cells rely on a highly ordered vesicle transport system to transfer membranes and proteins between different intracellular compartments. A number of proteins have been identified in transport vesicle targeting and fusion with the target membrane. From yeast to man, SNARE family proteins are essential for membrane fusion [1]. SNARE proteins can be divided in distinct subfamilies that all share helical regions with heptad repeats referred to as the SNARE motifs [2]. SNARE motifs from different SNARE proteins can interact with each other to form a dense helix bundle, the SNARE complex [3], [4], [5].

The formation of a SNARE complex is typically followed by membrane fusion. Syntaxin family SNARE proteins are integral membrane proteins that belong to Q-SNAREs i. e. they contain a glutamine at the central layer of the SNARE motif bundle [3], [5]. In addition to the SNARE motif, syntaxins have an N-terminal domain that is composed of three short helixes and a C-terminal transmembrane domain that is followed by a very short hydrophilic tail [5], [6]. *S. cerevisiae* expresses two highly homologous syntaxins Sso1p and Sso2p that both mediate membrane fusion during exocytosis at the plasma membrane [7].

The Sso1p the N-terminal domain has been shown to interact with the SNARE motif and regulate the rate of SNARE complex assembly [8]. Together, Sso1p and Sso2p perform an essential function in vegetatively growing haploid and diploid cells [7] where they interact with plasma membrane SNARE proteins Sec9p, Snc1p and Snc2p [9], [10]. However, in meiotic diploid cells there is a specific requirement for Sso1p for *de novo* formation of the prospore membrane during meiosis [11], [12], [13], [14].

The functional difference for Sso1p and Sso2p in meiotic cells is not explained by transcriptional regulation, or differences in expression levels. Both proteins are expressed at similar level in meiotic cells, localize to the prospore membrane, and swapping of promoters between *SSO1* and *SSO2* does not render Sso2p functional in prospore membrane formation [11], [15], [16]. The two N-terminal α-helices Ha and Hb of Sso1p are important for its function during meiosis [16]. In addition to the specific requirement of Sso1p, in sporulating cells the Q-SNARE Sec9p is replaced by a homologous protein Spo20 [17], [18], [19]. Recent results indicate that phosphatidic acid and PI(4,5)P_2_ are important for membrane fusion during prospore membrane formation [15]. However, the signals that regulate the activity of Sso1p and the initiation of meiotic SNARE complex formation are unknown.

Post-translational modifications are central modifiers of protein activity [20], [21]. Mass spectrometry studies have revealed *in vivo* phosphorylation sites in the amino terminal part of Sso1p and Sso2p [22]. In this study we set out to establish the contribution of these phosphoamino acids on the functional regulation of Sso1 and Sso2 proteins. In addition, we tested, whether, in analogy to meiosis and sporulation, also pseudohyphal and invasive growth, two nutritionally regulated cell differentiation processes display differential requirements for Sso1p and Sso2p.

## Results and Discussion

### Sso1 and Sso2 Phosphorylation

**Figure 1:**
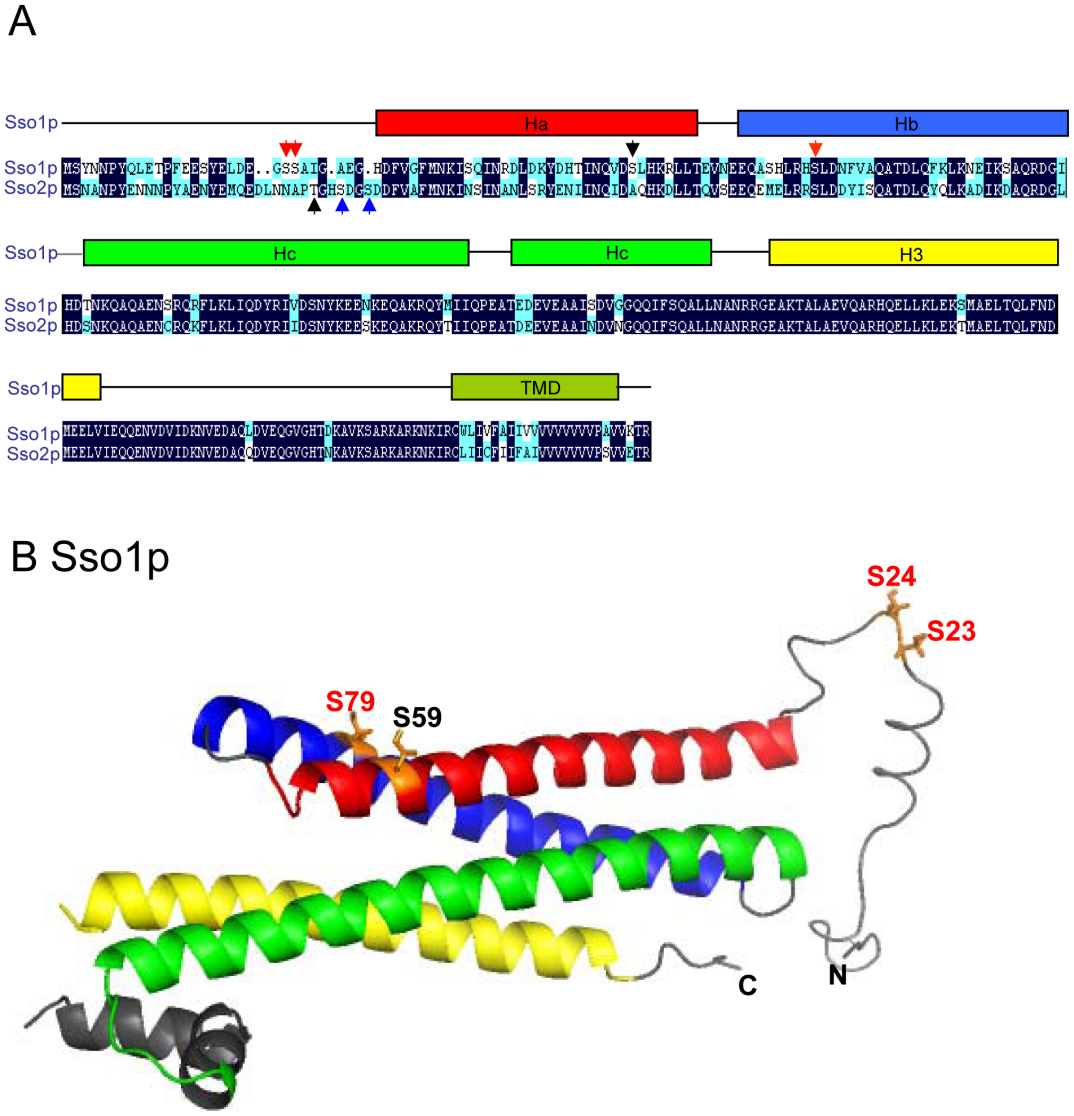
A schematic diagram illustrating the Sso1p and Sso2p homology and the domain structure of Sso1p. A) Habc, H3 SNARE motif, and transmembrane domain (TMD) are indicated. Serine 23, serine 24, serine 79 in Sso1p (red arrows) and serine 31 and serine 34 in Sso2p (blue arrows) indicate the identified *in vivo* phosphorylation sites [22], [23]. Additional amino acids mutagenized (Serine 59 in Sso1p and Threonine 28 in Sso2p) are indicated by black arrows. B) The three dimensional structure of Sso1p (PDB 1FIO, [8]) with an added random N-terminal peptide for amino acids 1–30. For the phosphoamino acids the side chains are shown. Phosphorylation sites identified by mass spectrometry or *in vivo* labeling are indicated by red colour. The additional amino acid mutagenized (Serine 59) is indicated by black colour.

Sso1p and Sso2p are highly homologous (75% identical, 88% similarity) (Figure 1A). Despite their similarity, only Sso1p, but not Sso2p is functional in prospore membrane formation in meiotic diploid cells [11], [16]. This suggests that mechanisms exist that enable cells to discriminate between these two homologous Q-SNARE proteins for SNARE complex formation in meiotic diploid cells. Recent analysis of *S. cerevisiae* phosphoproteome has identified serines 23 and 24 in Sso1p and serines 31 and 34 in Sso2p as *in vivo* phosphorylation sites [22]. In addition, serine 79 was previously reported as an *in vivo* phosphorylation site in Sso1p [23]. Subsequent analysis showed that S79 phosphorylation reduced participation of Sso1p in haploid cell SNARE complexes [23]. These amino acids (Figure 1A) represent potential regulatory means to modulate Sso protein *in vivo* function and differentiate between these proteins during sporulation.

The structure of a cytosolic fragment of Sso1p (amino acids 31–225) has been determined [8]. This structure is missing the very amino-terminus that contains several phosphorylation sites in Sso1p and the homologous Sso2p. The amino-terminal peptides of several syntaxins do not refract well in crystals. This suggests that even when present in the analyzed protein the peptide is unstructured in monomeric syntaxins. In order to better visualize the localization of the indicated *in vivo* phosphorylation sites a random peptide model was generated for the amino-terminal peptide of Sso1p (Figure 1B). When the putative phosphoamino acids were displayed in this model and the known structure, it is evident that Sso1p S23, S24 and S79 are located either in the Hb helix or at the unstructured amino-terminal peptide. At both locations, they are apparently accessible for cytosolic interactions.

### Sso1p and Sso2p Phosphorylation Mutants are Functional *In Vivo*

In order to assess the functionality of Sso1p and Sso2p phosphorylation sites the putative phosphoamino acids S23, S24 and S79 in Sso1p and S31 and S34 in Sso2p were mutagenized to alanine or glutamic acid to mimic either constitutively non-phosphorylated or phosphorylated forms of these amino acids. These mutant *sso1* and *sso2* genes were cloned to centromeric low copy and to 2 µ high copy vectors. In both vectors the expression of *SSO* genes was maintained under the endogenous *SSO1* and *SSO2* promoters, respectively. In order to test the functionality of these mutant proteins in cells where they were the only Sso proteins expressed, these plasmids were transformed into the *GAL1-SSO1 sso1Δ sso2Δ* cells (H3664) where the wt *SSO1* expression can be shut down by shifting cells from galactose containing medium to glucose containing medium. In glucose medium cells transformed with the empty vector ceased to grow (Table 1 and Table 2). However, no difference in growth, even at high temperatures, was observed for cells expressing either the mutant versions or the wt *SSO1* or *SSO2* at low or high levels (Table 1 and Table 2).

**Table 1:** Complementation capacity of *sso* and *sec* mutants.

Mutants	24°C	30°C	34°C	37°C	38°C
***GAL1-SSO1 sso1Δ sso2Δ***					
**Sso1 S59A**	+	+	+	+	+
**Sso1 S59E**	+	+	+	+	+
**Sso1 S59A S79A**	+	+	+	+	+
**Sso1 S59E S79E**	+	+	+	+	+
**Sso1 S23A S24A**	+	+	+	+	+
**Sso1 S23E S24E**	+	+	+	+	+
**Sso1 S23A S24A S59A**	+	+	+	+	+
**Sso1 S23E S24E S59E**	+	+	+	+	+
**Sso1 S23A S24A S59A S79A**	+	+	+	+	+
**Sso1 S23E S24E S59E S79E**	+	+	+	+	+
**Sso1 wt**	+	+	+	+	+
**Sso2 T28A S31A S34A**	+	+	+	+	+
**Sso2 T28E S31E S34E**	+	+	+	+	+
**Sso2 wt**	+	+	+	+	+
**Vector**	−	−	−	−	−
***sso1 Δsso2-1***					
**Sso1 S59A**	+	+	+	+	nd
**Sso1 S59E**	+	+	+	+	nd
**Sso1 S59A S79A**	+	+	+	+	nd
**Sso1 S59E S79E**	+	+	+	+	nd
**Sso1 S23A S24A**	+	+	+	+	nd
**Sso1 S23E S24E**	+	+	+	+	nd
**Sso1 S23A S24 S59A**	+	+	+	+	nd
**Sso1 S23E S24E S59E**	+	+	+	+	nd
**Sso1 S23A S24A S59A S79A**	+	+	+	+	nd
**Sso1 S23E S24E S59 S79E**	+	+	+	+	nd
**Sso1 wt**	+	+	+	+	nd
**Vector**	+	−	−	−	nd
***sso1-1 sso2Δ***					
**Sso2 T28A S31A S34A**	+	+	+	+	**+**
**Sso2 T28E S31E S34E**	+	+	+	+	**+**
**Sso2 wt**	+	+	+	+	**+**
**Vector**	+	+	+	+	**−**

nd, not determined.

**Table 2:** Multicopy suppression capacity of *sso* mutants.

Mutants	24°C	28°C	30°C	31°C	32°C	33°C	34°C	35°C	36°C	37°C
***GAL1-SSO1 sso1Δ sso2Δ***										
**Sso1 S59A**	+	nd	+	nd	nd	nd	+	nd	nd	+
**Sso1 S59E**	+	nd	+	nd	nd	nd	+	nd	nd	+
**Sso1 S59A S79A**	+	nd	+	nd	nd	nd	+	nd	nd	+
**Sso1 S59E S79E**	+	nd	+	nd	nd	nd	+	nd	nd	+
**Sso1 S23A S24A**	+	nd	+	nd	nd	nd	+	nd	nd	+
**Sso1 S23E S24E**	+	nd	+	nd	nd	nd	+	nd	nd	+
**Sso1 S23A S24A S59A**	+	nd	+	nd	nd	nd	+	nd	nd	+
**Sso1 S23E S24E S59E**	+	nd	+	nd	nd	nd	+	nd	nd	+
**Sso1 S23A S24A S59A S79A**	+	nd	+	nd	nd	nd	+	nd	nd	+
**Sso1 S23E S24E S59E S79E**	+	nd	+	nd	nd	nd	+	nd	nd	+
**Sso1 wt**	+	nd	+	nd	nd	nd	+	nd	nd	+
**Sso2 T28A S31A S34A**	+	nd	+	nd	nd	nd	+	nd	nd	+
**Sso2 T28E S31E S34E**	+	nd	+	nd	nd	nd	+	nd	nd	+
**Sso2 wt**	+	nd	+	nd	nd	nd	+	nd	nd	+
**Vector**	−	nd	−	nd	nd	nd	−	nd	nd	−
***sso1 Δsso2-1***										
**Sso1 S79A**	+	+	+	+	+	+	−	−	−	−
**Sso1 S79E**	+	+	+	+	+	+	−	−	−	−
**Sso1 S23A S24A S59A S79A**	+	+	+	+	+	+	−	−	−	−
**Sso1 S23E S24E S59 S79E**	+	+	+	+	+	+	−	−	−	−
**Sso1 wt**	+	+	+	+	+	+	−	−	−	−
**Vector**	+	+	+	+	+	−	−	−	−	−
***sso1-1 sso2Δ***										
**Sso2 T28A S31A S34A**	+	nd	+	nd	nd	nd	+	+	+	**+**
**Sso2 T28E S31E S34E**	+	nd	+	nd	nd	nd	+	+	+	**+**
**Sso2 wt**	+	nd	+	nd	nd	nd	+	+	+	**+**
**Vector**	+	nd	+	nd	nd	nd	+	+	+	**−**

nd, not determined.

**Figure 2:**
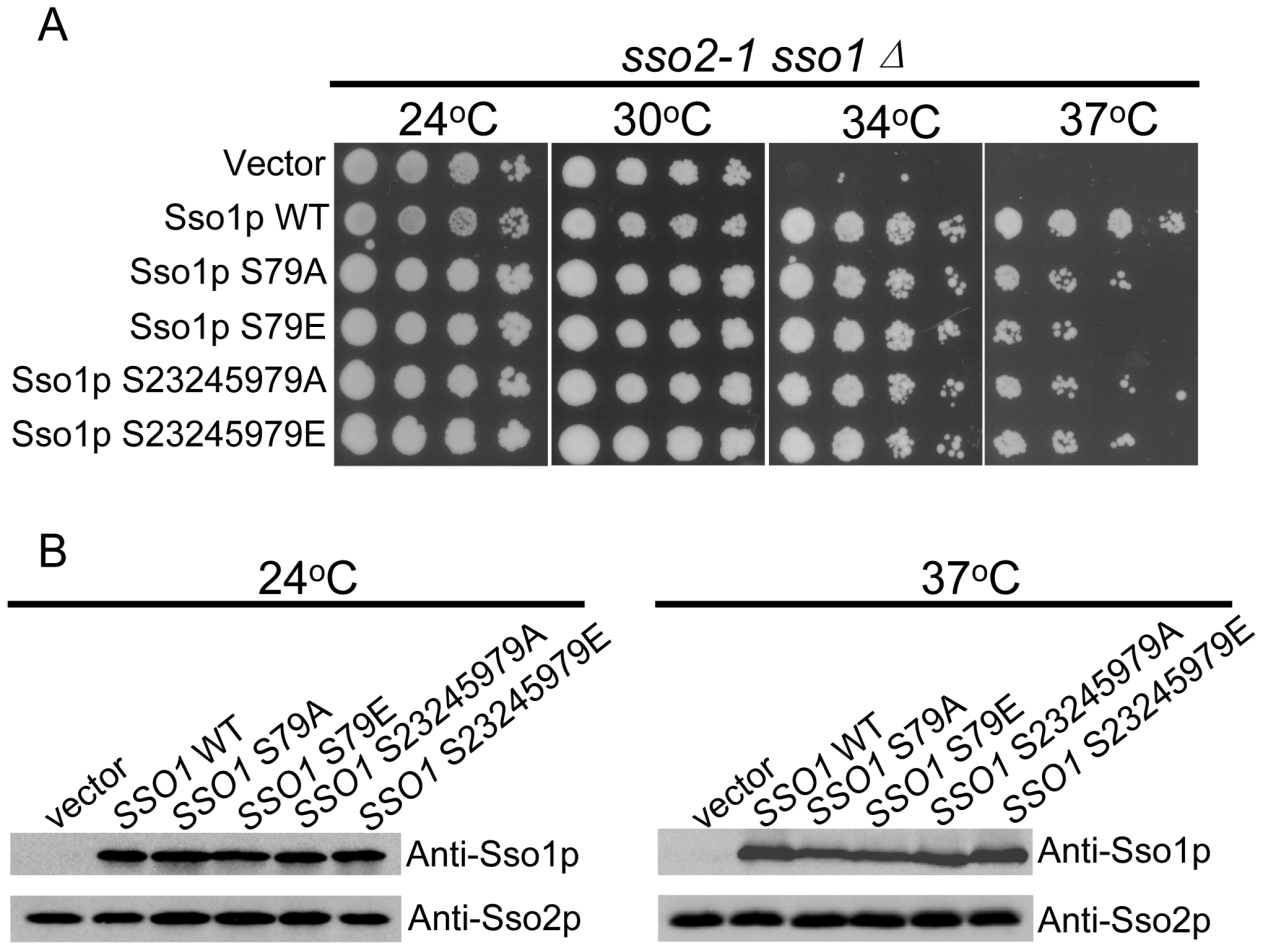
Serine to alanine (A) or glutamic acid (E) single mutation or combined do not inactivate Sso1p or Sso2p *in vivo*. A) The growth of serial 10-fold dilutions of *sso1Δ sso2-1* cells expressing different mutant variants of Sso1p from a low copy plasmid at different temperatures. B) The mutant proteins generated are expressed at similar levels in *S. cerevisiae* cells. The *sso1Δ sso2-1* cells (H2177) expressing different *sso1* mutants were grown at designed temperatures and subjected to SDS-PAGE and Western blotting with anti-Sso1p or anti-Sso2p specific antibodies.

In order to assess the mutant protein functionality in a different way, the temperature-sensitive *sso1Δ sso2-1* (H2177) yeast strain was transformed with plasmids for expression of the mutant Sso1p or Sso2p or the empty vector as a control. The ability of the mutant versions of Sso1p and Sso2p to rescue the temperature-sensitivity of this strain was scored (Figure 2, Table 1 and Table 2). Previously, phosphorylation of S79 was shown to reduce the recruitment of Sso1p to exocytic SNARE complexes in haploid yeast cells [23]. The *sso1(S79A)* mutant overexpression could efficiently rescue rich medium sensitivity of *snc1Δ snc2Δ* cells [23]. In that study, the ability of the phosphorylation mimicking S79E/D mutant was not tested.

When compared to the wild type *SSO1* (expressed from a centromeric, low copy plasmid) both *sso1(S79A)* and *sso1(S79E)* were slightly less efficient in rescuing the temperature-sensitivity of *sso2-1 sso1Δ* (H2177) cells (Figure 2A). Repeatedly, *sso1(S79E)* was slightly less efficient than *sso1(S79A)* in its suppression capacity in *sso2-1 sso1Δ* cells. However, when all the identified phosphoamino acids in Sso1p or Sso2p were mutagenized separately or simultaneously to alanines or glutamic acids, no additional phenotype over the S79E or S79A was observed (Table 1). Furthermore, when compared to overexpression of wt *SSO1* or *SSO2*, overexpression of the mutant versions of *sso1* or *sso2* did not result in additional growth phenotypes (Table 2).

It has been shown that S and T phosphorylation sites cluster [24]. At the same time cells can use processive phosphorylation of adjacent amino acids to modulate the strength or threshold of the responses [25]. Although, the sensitivity of mass spectrometry is high it is possible that not all phoshopeptides are resolved with the current methods. Because the already generated mutations did not significantly affect Sso1p or Sso2p activity, additional mutations were generated in Sso1p (Serine 59) and Sso2p (Threonine 28) that locate adjacent to the identified phosphoamino acids (Figure 1A and B). When tested for complementation or multicopy suppression of the defective Sso1p or Sso2p, the functionality of mutant proteins containing all mutations was comparable to that of the wt proteins *in vivo* (Figure 2A, Table 1). Similarly, these mutants were able to complement and multicopy supppress the temperature-sensitive phenotype of *sso1-1 sso2Δ* (H2608) cells (Table 2). All *sso1* and *sso2* mutants were expressed at similar expression levels irrespective of the growth temperature of the cells (Figure 2B). This indicates that introduction of these mutations does not affect the stability of Sso1p and Sso2p. In addition, this indicates that the observed minor defect in complementation of *sso2-1 sso1Δ* cell temperature-sensitivity is not due to reduced expression levels.

Previously, high copy expression of *SSO1* or *SSO2* was shown to suppress *sec1-1*, *sec9-4* and *sec15-1* mutant cell growth defect at the restrictive temperature [7]. The mutants generated here were as efficient as the wild type *SSO1* and *SSO2* to rescue *sec1-1*, *sec9-4* and *sec15-1* growth defect at the restrictive temperature (data not shown). Collectively, our results show that phosphorylation or dephosphorylation of the tested amino acids in Sso1p or Sso2p is not essential for vegetative growth of haploid *S. cerevisiae* cells. At the same time, these mutants had no detectable dominant negative effects on cell growth. However, in line with the previous results reporting a role for Sso1p S79 phosphorylation in Sso1p regulation [23], Sso1pS79E and Sso1pS79A mutants were not fully as effective as the wt Sso1p in complementing the temperature-sensitive growth of the *sso2-1 sso1Δ* cells.

### Phosphorylation Mutants of Sso1p Do Not Affect Sporulation

*SSO1*, but not *SSO2* is essential for prospore membrane formation [11]. We used this essential function of Sso1p to map possible contribution of the identified phosphoamino acids for Sso1p function in this cell differentiation process. For this *sso1Δ/sso1Δ* diploid cells were generated where different mutant versions of *sso1* (expressed from endogenous *SSO1* promoter) were integrated at the *ura3–52* locus. For each mutant, three independent transformants were induced to sporulate synchronously. The formation of spores was quantified by counting cells that were able to form tetrads (Table 3). The results show that mutations in the tested amino acids in Sso1p do not affect Sso1p functionality in prospore membrane formation. That no sporulation phenotype was observed in these Sso1p mutants was surprising given the fact that S79A alone has been shown to affect SNARE complex assembly [23].

**Table 3:** Quantification of Tetrads In *sso1* Mutants.

Mutant	tetrads	no tetrads	total	tetrad %
Sso1p S79A	343	78	421	81
Sso1p S79E	374	78	452	83
Sso1p S23A S24A S59A S79A	316	72	388	81
Sso1p S23E S24E S59E S79E	293	70	363	81
Sso1p wt	293	66	359	82
Vector	0	395	395	0

In prospore membrane formation Sso1p forms complexes with a Sec9p homologue Spo20p and Snc2p to drive membrane fusion [17], [18]. Our results suggest that in meiotic diploid cells the prospore membrane formation is not critically sensitive to S79 phosphorylation. We can not exclude the possibility that there are additional amino acids that have phospho- or some other post-translational modifications that regulate Sso protein function. The mechanism how *S. cerevisiae* cells can selectively use Sso1p, instead of the highly homologous Sso2p, for membrane fusion in meiotic diploid cells, remains enigmatic. We have previously identified Mso1p as an essential protein for prospore membrane formation [26]. Mso1p exists in complex with Sec1p, a regulator of SNARE complex assembly [26], [27]. Interestingly, Mso1p binds preferentially Sso1p [27]. Interactions with Mso1p may provide additional specificity for the selective activity of Sso1p in prospore membrane formation.

### Haploid or Diploid Cell Pseudohyphal or Invasive Growth are Not Differentially Regulated by *SSO1* or *SSO2*

When starved for nitrogen, diploid cells undergo a developmental transition from a single cell yeast form to a filamentous pseudohyphal form [28]. Pseudohyphal filaments are composed of chains of elongated cells that radiate away from the colony and penetrate the agar substratum on which they are grown [28], [29]. In this process changes in cell polarity and budding mode take place. Although poorly understood, it is conceivable that changes in cell polarity and budding mode involve regulation of protein and membrane transport to the plasma membrane and thus require the activity of Sso1p and/or Sso2p. Different types of pseudohyphal growth are observed in *S. cerevisiae* cells. In addition to the originally identified nitrogen starvation triggered diploid cell differentiation process [28], subsequent studies have shown that both haploid and diploid cells can be induced to form short branched pseudohyphae in liquid cultures in response to “fusel” alcohols such as 1-butanol [30].

**Figure 3:**
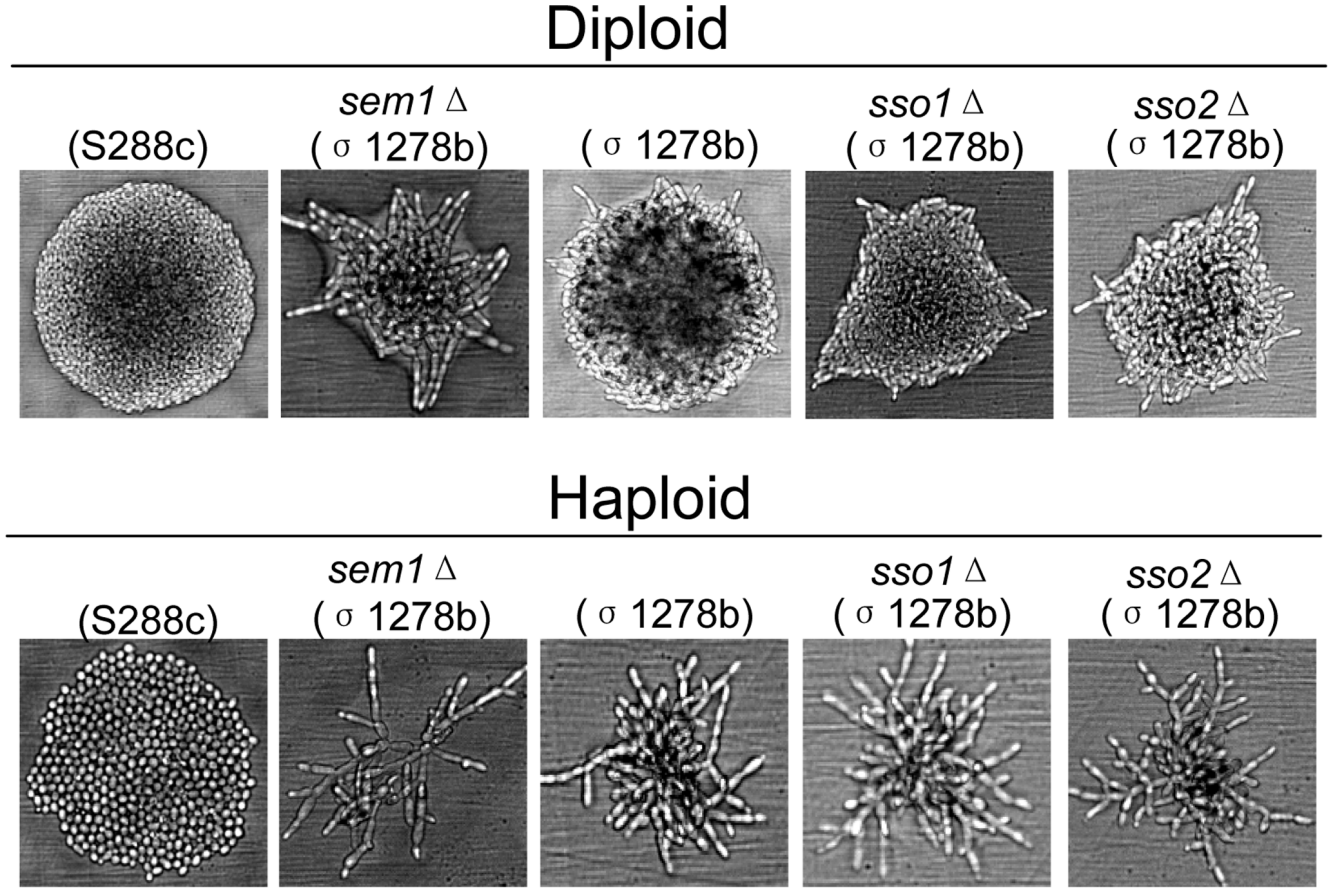
Diploid and haploid pseudohyphal growth is not affected by deletion of either *SSO1* or *SSO2*. Upper panel: Diploid cells were streaked on (synthetic low-ammonia dextrose) SLAD medium and incubated for one day in order to examine the morphology of the colonies. Lower panel: Haploid cells were streaked on YPD medium supplemented with 1% (v/v) 1-butanol and incubated for one day before examination of the colony morphology. Negative control cells of S288C background haploid (H973) and diploid (H1700) devoid of ability to form pseudohyphae. Positive controls for haploid (H2186) and diploid (H3088) cells of the Σ1278b background where a negative regulator of pseudohyphal growth (*SEM1*) was deleted [32].

**Figure 4:**
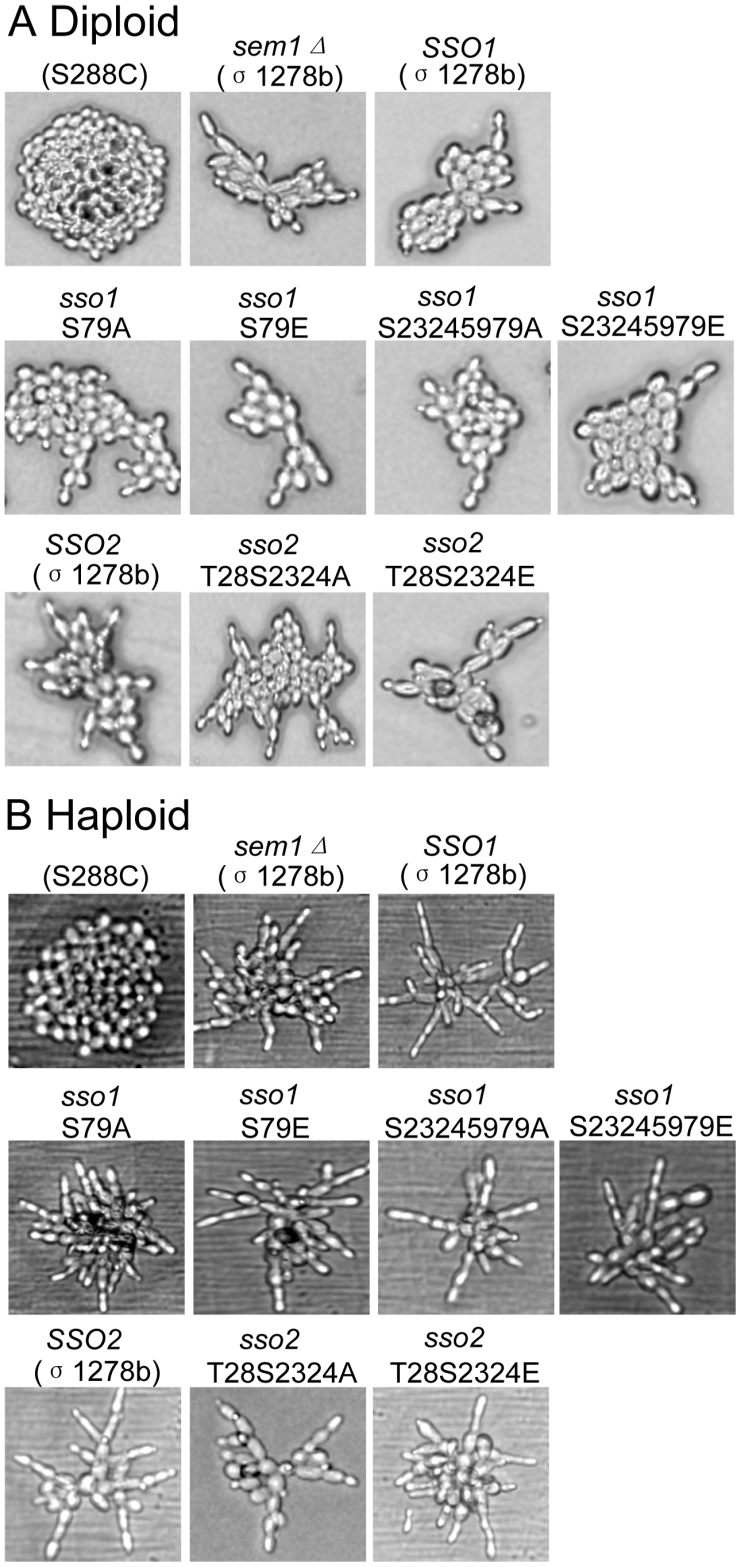
Haploid and diploid pseudohyphal growth is not affected by mutations in the putative phosphoamino acids in Sso1p or Sso2p. A) Diploid cells expressing as their sole copy of *SSO* either the wt *SSO1* (H3970), *SSO1S79A* (H3966), *SSO1 S79E* (H3967), *SSO1S23245979A* (H3968) or *SSO1S23245979E* (H3969), or the wt *SSO2* (H3973), *SSO2T28S3134A* (H3971) or *SSO2T28S3134E* (H3972). B) Haploid cells expressing as their sole copy of *SSO* either the wt *SSO1* (H3959), *SSO1S79A* (H3955), *SSO1 S79E* (H3956), *SSO1S23245979A* (H3957) or *SSO1S23245979E* (H3958), or the wt *SSO2* (H3965), *SSO2T28S3134A* (H3963) or *SSO2T28S3134E* (H3964). Treatment of cells as described in Figure 3. The negative control was for Haploid (H973) and diploid (H3088) cells of S288C background. Positive controls for haploid (H2186) and diploid (H3088) cells of the Σ1278b background where a negative regulator of pseudohyphal growth (*SEM1*) was deleted [32].

In order to assess the possible specific roles of *SSO1* or *SSO2* in nitrogen starvation or alcohol induced pseudohyphal growth, *SSO1* and *SSO2* were deleted both in the haploid and diploid cells of Σ1278b background widely used in studies concerning pseudohyphal growth. In addition, as a negative control haploid and diploid cells of S288c background were tested for pseudohyphal growth. Previously, S288c cells were shown to be defective for pseudohyphal growth due to a mutation in *FLO8* [31]. As a positive control for pseudohyphal growth Σ1278b cells deleted for *SEM1* were used. Previously, deletion of *SEM1* was shown to enhance pseudohyphal growth [32]. Homozygous diploid cells deleted either for *SSO1* or *SSO2* were capable of forming pseudohyphae on low nitrogen SLAD plates (Figure 3, upper panel). Similarly, haploid cells deleted either for *SSO1* or *SSO2* formed extensive hyphae on YPD plates supplemented with 1% 1-butanol (Figure 3, lower panel). In order to test the possible contribution of phosphorylation on Sso1p and Sso2p activity during pseudohyphal growth, haploid and diploid cells (Σ1278b background) were generated that express as their sole copy of Sso proteins the phospho-mutant versions of Sso1p or Sso2p. Microscopic analysis of these cells revealed that Sso1p(S23S24S59S79) or Sso2p(T28S31S34) alanine or glutamic acid mutations display no obvious defect in pseudohyphal growth (Figure 4).

**Figure 5:**
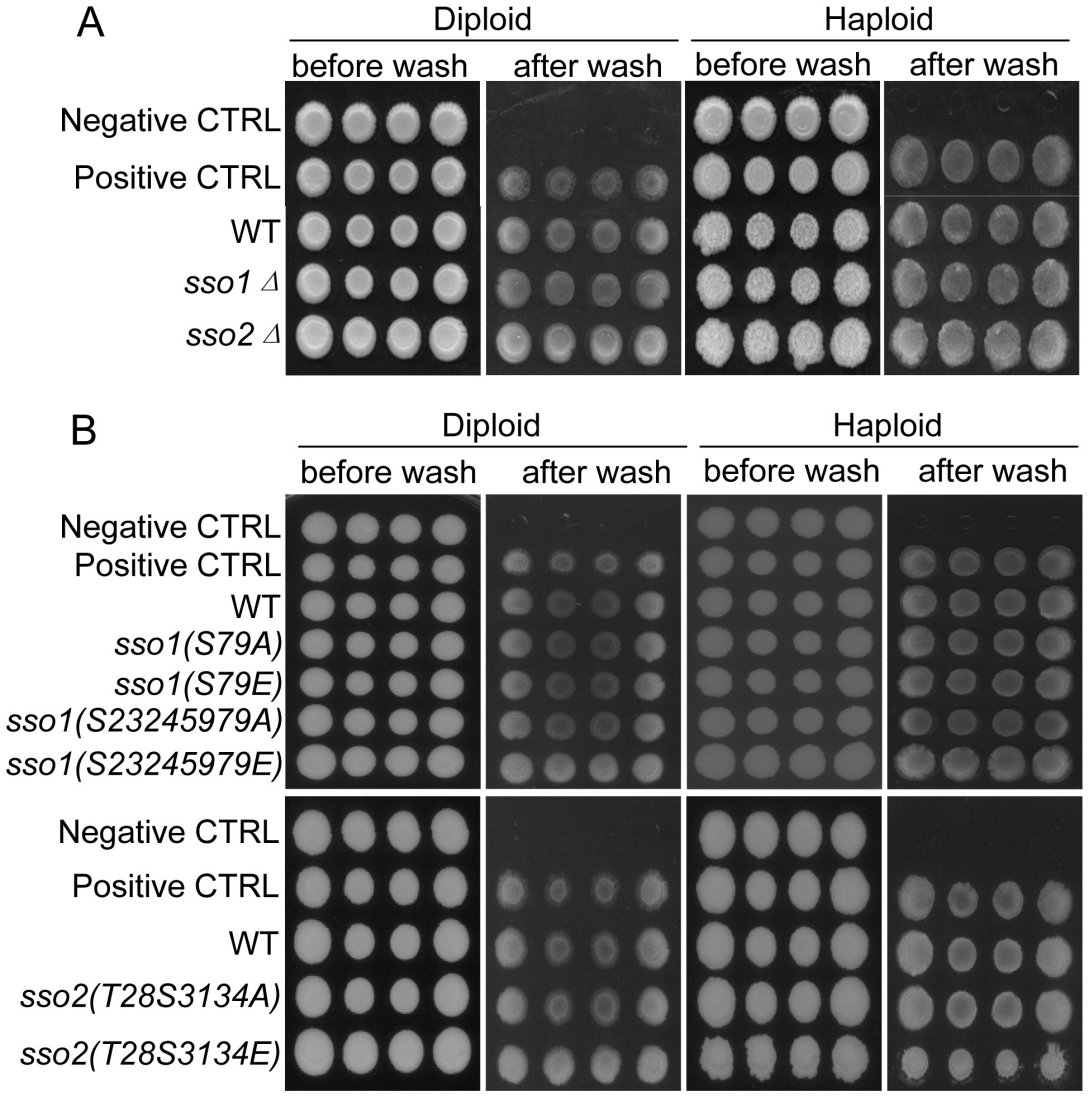
*SSO1* or *SSO2* are not required for, and phosphomutations in Sso1p or Sso2p do not affect, diploid or haploid cell invasive growth. Patches of cells from four independent colonies were grown at 30°C for 3 days and incubated at room temperature for an additional 2 days. Non-invasive cells were rinsed away with a gentle stream of deionized water from the agar surface. A) Invasive growth of cells deleted either for *SSO1* (diploid H3843), (haploid H3839) or *SSO2* (diploid H3845), (haploid H3841). B) Upper panel: Invasive growth of cells expressing as their sole copy of *SSO* genes the wt *SSO1* (diploid H3970), (haploid H3959) the phosphomimicking *SSO1S79E* (diploid H3967), (haploid H3956), *SSO1S23245979E* (diploid H3969), (haploid H3958) or the putative non-phosphorylated *SSO1S79A* (diploid H3966), (haploid H3955), *SSO1S23245979A* (diploid H3968), (haploid H3957). Lower panel: Invasive growth of cells expressing as their sole copy of *SSO* genes the wt *SSO2* (diploid H3973), (haploid H3965) or the phosphomimicking *SSO2T28S3134E* (diploid H3972), (haploid H3964) or the putative non-phosphorylated *SSO2T28S3134A* (diploid H3971, (haploid H3963). In A) and B) cells of S288c background were used as negative, non-invasive haploid (H973) and diploid (H1700) controls. Positive controls for haploid (H2186) and diploid (H3088) invasive growth were cells of Σ1278b background where a negative regulator of pseudohyphal growth (*SEM1*) was deleted [32].

On solid growth medium (agar) both haploid and diploid cells of Σ1278b background display invasive growth [30]. To assess whether *SSO1* or *SSO2* are specifically involved in the regulation of invasive growth, *SSO1* or *SSO2* deleted cells expressing the phospho-mutant versions of Sso1p or Sso2p as their sole copy of Sso proteins (in Σ1278b background) were tested for invasive growth. For this, equal amounts of (OD_600_ 1) haploid and diploid cells of four independent colonies were spotted on YPD plates. Cells were allowed to grow at 30°C for 3 days followed by incubation at room temperature for two additional days [30]. The plates were rinsed with a gentle stream of deionized water to remove non-invaded cells. As shown in Figure 5A, deletion of *SSO1* or *SSO2* in haploid *MAT*α (or *MAT* **a**, data not shown) or diploid cells had no effect on invasive growth. Similarly, the phosphoamino acid mimicking or abolishing mutations in Sso1p or Sso2p had no obvious effect on the ability of cells to invade the agar (Figure 5B). At the same time, the non-invasive control strain S288c (Figure 5, negative ctrl) was unable to invade the agar and the cells were easily washed away.

Collectively, our results suggest that differential participation of Sso1p and Sso2p in membrane fusion during a nutrient triggered cell differentiation process is not a general mode of regulation for cell growth. In addition, our results show that the currently identified phosphoamino acids are not essential for Sso1p or Sso2p function *in vivo*. This could be due to differential targeting of downstream factors by different nutrient triggered signaling events in membrane fusion during sporulation and pseudohyphal growth. Alternatively, it is possible that protein phosphorylation is not a decisive event in membrane fusion regulation in these cellular processes or that additional, currently uncharacterized phosphorylation sites or other post-translational modifications exist in Sso1p and Sso2p.

## Materials and Methods

### Yeast strains

The yeast strains used are listed in Supplementary Table S1. When not stated otherwise, standard growth media were used [33]. *LEU2* and *LYS2* were deleted in H1925 and H1926 by transforming the cells with *Sal*I cut pAD1 or *Cla*I cut pAD2 [34]. Ura+ colonies were patched on SC-ura, replica-plated to YPD (to enable loss of the *URA3* marker and flanking vector sequences) and replica-plated to 5-FOA plates. Papillae from the 5-FOA plates were streaked onto YPD and replica-plated either to SC-leu or SC-lys to identify the desired auxotrophic mutants. *SSO1* and *SSO2* were deleted with kanMX by using the PCR cassette based transformation method [35]. The sequence informatiom of the oligonucleotides used in this study is available upon request. The deletions were verified both by PCR and by Western blotting with Sso1p and Sso2p specific antibodies [11]. Diploid strains were obtained by mating of appropriate haploid cells. In order to test the functionality of different *sso1* mutants in the diploid *sso1Δ/sso1Δ* strain during sporulation, H3114 was transformed with integrative plasmids linearized by a *Stu*I cut within the *URA3* and selected for growth at 24°C in the absence of uracil. For pseudohyphal growth, plasmids expressing wt or mutant versions of *SSO1* or *SSO2* were integrated to haploid cells (H3836, H3837, H3839 and H3841) where either the *SSO1* or *SSO2* had previously been deleted. In the resulting integrants (H3960, H3961, H3962, H3963, H3964 and H3965) either *SSO1* or *SSO2* was then deleted using either kanMX or hphNT1 containing PCR cassettes [35]. Appropriate haploid cells were then mated to generate homozygous diploids where the mutant versions of *SSO1* or *SSO2* were the sole copy of *SSO* genes. The obtained diploid strains were verified for expression of *SSO1* or *SSO2* by Western blotting with Sso1p and Sso2p specific antibodies [11].

### Plasmids

*SSO1* genomic fragment (453 bp upstream of ATG and 501 bp downstream of stop) in B1473 was mutagenized using the QuickChange method (Stratagene) to generated S23, S24, S59, S79 mutations to alanine or glutamic acid. The mutagenized genes were sequenced and cloned as *BamH*I-*EcoR*I fragments into pRS406, pRS416 and pRS426. Using B1474 as a template, the genomic *SSO2* fragment (435 bp upstream of ATG and 1005 bp downstream of stop) was similarly mutagenized to change T28 and S31 and S34 to alanine or glutamic acid. The mutagenized genes were sequenced and cloned as *BamH*I-*EcoR*I fragments into pRS406, pRS416 and pRS426.

### Complementation and Suppression Tests for Temperature-sensitive Growth

The complementation or multicopy suppression of the temperature-sensitive growth phenotypes was assayed by transforming plasmids expressing the wild type, mutant versions of *SSO1* or *SSO2* or the empty vector to *sso2Δ GAL1-SSO1* strain (H3664) or to *sso* mutant strains H2177 and H2608. In case of H3664 cells were grown on SC-ura 2% galactose followed by replication to SC-ura 2% glucose at different temperatures. Alternatively, plasmids were transformed to mutant strains *sec1-1* (H305), *sec9-4* (H3860) and *sec15-1* (H761). Initially, patches of four independent transformants were tested for growth at different temperatures on SC-ura plates for three days. Finally, ten-fold dilution series of OD_600_ 1 cells were generated, dotted on a SC-ura plates and their growth was monitored for three days.

### Yeast Cell Lysates

For evaluation of Sso1p and Sso2p mutant protein expression levels, cells were grown to OD_600_ 1, the cultures were split into two identical halves and grown either at 24°C or 37°C for another 2 h. Cells were broken by vortexing in the presence of 0.45 mm glass beads in 2% SDS supplemented with a protease inhibitor cocktail (Complete, Roche). Lysates were centrifuged for 10 minutes at 20,200 g followed by heating of supernatants for 5 minutes at 95°C. The protein concentration was determined with BCA™ Protein Assay Kit (Thermo scientific). Equal amount of total protein from each lysate was subjected to 12% SDS-polyacrylamide gel and analyzed by Western blotting using anti-Sso1p and anti-Sso2p specific antibodies [11].

### Liquid Sporulation

Cells were grown overnight in YPD (with 5% glucose) diluted to OD_600_ 0.1 in 1% KAc, 2% peptone, 1% yeast extract (presporulation medium) and grown at 30°C overnight. Cells were washed once with water and resuspended to OD_600_ 1 in 1% KAc. The development of tetrads was monitored by microscopy. The tetrads were counted using hemocytometer (Assistent, Germany).

### Pseudohyphal and Invasive Growth

Diploid cell pseudohyphal growth was tested on SLAD plates [28] supplemented with appropriate amino acids at 30°C for 1 day followed by examination with Olympus AX 70 Microscope. Haploid cell pseudohyphal growth was induced on YPD plates supplemented with 1% (v/v) 1-butanol at 30°C for 1 day, and then monitored by microscopy [30]. Invasive growth was tested by spotting equal amount of OD_600_ 1 cells on YPD plates. Cells were allowed to grow at 30°C for 3 days followed by incubation at room temperature for two additional days. The plates were rinsed with a gentle stream of deionized water to remove non-invaded cells and photographed.

## Supporting Information

Table S1 Yeast strains.
(0.12 MB DOC)
