# Supplementary material for: Functional Analysis of Phosphorylation on Saccharomyces cerevisiae Syntaxin 1 Homologues Sso1p and Sso2p
Source: PLoS One. 2010 Oct 11;5(10):e13323. doi: 10.1371/journal.pone.0013323 (PMC2952630; doi:10.1371/journal.pone.0013323)
Supplement: Table S1 — Yeast strains. (0.12 MB DOC) [file pone.0013323.s001.doc]

**Supplementary Table 1. Yeast Strains**

| Strain | Genotype | Source |
| --- | --- | --- |
| H304 | *MAT***a** *leu2-3,112 ura3-52* | Peter Novick |
| H973 | *MAT*α *his4-619 ura3-52* | Peter Novick |
| H1700 | *MAT***a** */ MAT*α *leu2-3, 112/LEU2 ura3-52/ura3-52 HIS4/his4-619* | Jäntti lab collection |
| H1925 | *MAT***a** *ura3* | E. Dubois |
| H1926 | *MAT*α *ura3* | E. Dubois |
| H2177 | *MAT***a** *sso2-1 ura3-52 leu2-3, 112 sso1::LEU2* | Hans Ronne [11] |
| H2185 | *MAT***a** *ura3 sem1::URA3* | Jäntti lab collection |
| H2186 | *MAT*α *ura3 sem1::URA3* | Jäntti lab collection |
| H2608 | *MAT*α *sso1-1 sso2::LEU2 ade2-1 his3-11,15 leu2-3,112 trp1-1 ura3-1 can1-100* | Hans Ronne [11] |
| H3088 | *MAT***a** */ MAT*α *ura3/ura3 sem1::URA3/sem1::URA3* | Jäntti lab collection |
| H3114 | *MAT***a** */ MAT*α *ura3/* *ura3 sso1::kanMX4/sso1::kanMX4 ho::hisG/ho::LYS2* | Hans Ronne [11] |
| H3664 | *MATα sso1-Δ1::LEU2 sso2-Δ1::LEU2::(GAL1-SSO1, HIS3) ade2-1* | Hans Ronne [11] |
|  | *his3-11,15 trp1-1 ura3-1 can1-100* |  |
| H3824 | *MAT***a** *ura3 lys2Δ0* | This study |
| H3825 | *MAT*α *ura3 leu2Δ0* | This study |
| H3831 | *MAT***a** */ MAT*α *ura3/ura3* | This study |
| H3836 | *MAT***a** *ura3 lys2Δ0 sso1Δ::kanMX4* | This study |
| H3837 | *MAT***a** *ura3 lys2Δ0 sso2Δ::kanMX4* | This study |
| H3839 | *MAT*α *ura3 leu2Δ0 sso1Δ::kanMX4* | This study |
| H3841 | *MAT*α *ura3 leu2Δ0 sso2Δ::kanMX4* | This study |
| H3843 | *MAT***a** */ MAT*α *ura3/ura3 sso1Δ::kanMX4/sso1Δ::kanMX4* | This study |
| H3845 | *MAT***a** */ MAT*α *ura3/ura3 sso2Δ::kanMX4/sso2Δ::kanMX4* | This study |
| H3950 | *MAT***a** *ura3::(YIp sso1 (S79A) URA3) lys2Δ0 sso1Δ::kanMX4 sso2Δ::hphNT1* | This study |
| H3951 | *MAT***a** *ura3::(YIp sso1 (S79E) URA3) lys2Δ0 sso1Δ::kanMX4 sso2Δ::hphNT1* | This study |
| H3952 | *MAT***a** *ura3::(YIp sso1(S23245979A) URA3) lys2Δ0 sso1Δ::kanMX4 sso2Δ::hphNT1* | This study |
| H3953 | *MAT***a** *ura3::(YIp sso1(S23245979E) URA3) lys2Δ0 sso1Δ::kanMX4 sso2Δ::hphNT1* | This study |
| H3954 | *MAT***a** *ura3::(YIp SSO1 wt URA3) lys2Δ0 sso1Δ::kanMX4 sso2Δ::hphNT1* | This study |
| H3955 | *MAT* α *ura3::(YIp sso1 (S79A) URA3) leu2Δ0 sso1Δ::kanMX4 sso2Δ::hphNT1* | This study |
| H3956 | *MAT*α *ura3::(YIp sso1 (S79E) URA3) leu2Δ0 sso1Δ::kanMX4 sso2Δ::hphNT1* | This study |
| H3957 | *MAT*α *ura3::(YIp sso1 (S23245979A) URA3) leu2Δ0 sso1Δ::kanMX4 sso2Δ::hphNT1* | This study |
| H3958 | *MAT*α *ura3::(YIp sso1( S23245979E) URA3) leu2Δ0 sso1Δ::kanMX4 sso2Δ::hphNT1* | This study |
| H3959 | *MAT*α *ura3::(YIp SSO1 wt URA3) leu2Δ0 sso1Δ::kanMX4 sso2Δ::hphNT1* | This study |
| H3960 | *MAT***a** *ura3::(YIp sso2 (T28S3134A) URA3) lys2Δ0 sso2Δ::kanMX4*  *lys2Δ0 sso2Δ::kanMX4* | This study |
| H3961 | *MAT***a** *ura3::(YIp sso2 (T28S3134E) URA3) lys2Δ0 sso2Δ::kanMX4* | This study |
| H3962 | *MAT***a** *ura3::(YIp SSO2 wt URA3) lys2Δ0 sso2Δ::kanMX4* | This study |
| H3963 | *MAT*α  *ura3::(YIp sso2 (T28S3134A) URA3) leu2Δ0 sso2Δ::kanMX4* | This study |
| H3964 | *MAT*α *ura3::(YIp sso2 (T28S3134E) URA3) leu2Δ0 sso2Δ::kanMX4* | This study |
| H3965 | *MAT*α *ura3::(YIp SSO2 wt URA3) leu2Δ0 sso2Δ::kanMX4* | This study |
| H3966 | *MAT***a***/MAT*α *ura3::(YIp sso1(S79A) URA3)/* *ura3::(YIp sso1(S79A) URA3)*  *ura3/ura3 leu2Δ0/LEU2 lys2Δ0/LYS2 sso1Δ::kanMX4/sso1Δ::kanMX4 sso2Δ::hphNT1/sso2Δ::hphNT1 [YIP sso1 S79A URA3]/[YIP sso1 S79A URA3] sso2Δ::hphNT1/sso2Δ::hphNT1 [YIP sso1 S79A URA3]/[YIP sso1 S79A URA3]* | This study |
|  | *leu2Δ0/LEU2 lys2Δ0/LYS2 sso1Δ::kanMX4/sso1Δ::kanMX4 sso2Δ::hphNT1/sso2Δ::hphNT1* |  |
| H3967 | *MAT***a***/MAT*α *ura3::(YIp sso1(S79E) URA3)/* *ura3::(YIp sso1(S79E) URA3)*  *ura3/ura3 leu2Δ0/LEU2 lys2Δ0/LYS2 sso1Δ::kanMX4/sso1Δ::kanMX4 sso2Δ::hphNT1/sso2Δ::hphNT1 [YIP sso1 S79A URA3]/[YIP sso1 S79A URA3] sso2Δ::hphNT1/sso2Δ::hphNT1 [YIP sso1 S79A URA3]/[YIP sso1 S79A URA3]* | This study |
|  | *leu2Δ0/LEU2 lys2Δ0/LYS2 sso1Δ::kanMX4/sso1Δ::kanMX4 sso2Δ::hphNT1/sso2Δ::hphNT1* |  |
| H3968 | *MAT***a***/MAT*α *ura3::(YIp sso1(S23245979A) URA3)/* *ura3::(YIp sso1(S232459S79A) URA3)*  *ura3/ura3 leu2Δ0/LEU2 lys2Δ0/LYS2 sso1Δ::kanMX4/sso1Δ::kanMX4 sso2Δ::hphNT1/sso2Δ::hphNT1 [YIP sso1 S79A URA3]/[YIP sso1 S79A URA3] sso2Δ::hphNT1/sso2Δ::hphNT1 [YIP sso1 S79A URA3]/[YIP sso1 S79A URA3]* | This study |
|  | *leu2Δ0/LEU2 lys2Δ0/LYS2 sso1Δ::kanMX4/sso1Δ::kanMX4 sso2Δ::hphNT1/sso2Δ::hphNT1* |  |
| H3969 | *MAT***a***/MAT*α *ura3::(YIp sso1(S23245979E) URA3)/* *ura3::(YIp sso1(S232459S79E) URA3)*  *ura3/ura3 leu2Δ0/LEU2 lys2Δ0/LYS2 sso1Δ::kanMX4/sso1Δ::kanMX4 sso2Δ::hphNT1/sso2Δ::hphNT1 [YIP sso1 S79A URA3]/[YIP sso1 S79A URA3] sso2Δ::hphNT1/sso2Δ::hphNT1 [YIP sso1 S79A URA3]/[YIP sso1 S79A URA3]* | This study |
|  | *leu2Δ0/LEU2 lys2Δ0/LYS2 sso1Δ::kanMX4/sso1Δ::kanMX4 sso2Δ::hphNT1/sso2Δ::hphNT1* |  |
| H3970 | *MAT***a***/MAT*α *ura3::(YIp SSO1 wt URA3)/* *ura3::(YIp SSO1 wt URA3)*  *ura3/ura3 leu2Δ0/LEU2 lys2Δ0/LYS2 sso1Δ::kanMX4/sso1Δ::kanMX4 sso2Δ::hphNT1/sso2Δ::hphNT1 [YIP sso1 S79A URA3]/[YIP sso1 S79A URA3] sso2Δ::hphNT1/sso2Δ::hphNT1 [YIP sso1 S79A URA3]/[YIP sso1 S79A URA3]* | This study |
|  | *leu2Δ0/LEU2 lys2Δ0/LYS2 sso1Δ::kanMX4/sso1Δ::kanMX4 sso2Δ::hphNT1/sso2Δ::hphNT1* |  |
| H3971 | *MAT***a***/MAT*α *ura3::(YIp sso2(T28S3134A) URA3)/* *ura3::(YIp sso2(T28S3134A) URA3)*  *ura3/ura3 leu2Δ0/LEU2 lys2Δ0/LYS2 sso1Δ::kanMX4/sso1Δ::kanMX4 sso2Δ::hphNT1/sso2Δ::hphNT1 [YIP sso1 S79A URA3]/[YIP sso1 S79A URA3] sso2Δ::hphNT1/sso2Δ::hphNT1 [YIP sso1 S79A URA3]/[YIP sso1 S79A URA3]* | This study |
|  | *leu2Δ0/LEU2 lys2Δ0/LYS2 sso1Δ::kanMX4/sso1Δ::kanMX4 sso2Δ::hphNT1/sso2Δ::hphNT1* |  |
| H3972 | *MAT***a***/MAT*α *ura3::(YIp sso2(T28S3134E) URA3)/* *ura3::(YIp sso2(T28S3134E) URA3)*  *ura3/ura3 leu2Δ0/LEU2 lys2Δ0/LYS2 sso1Δ::kanMX4/sso1Δ::kanMX4 sso2Δ::hphNT1/sso2Δ::hphNT1 [YIP sso1 S79A URA3]/[YIP sso1 S79A URA3] sso2Δ::hphNT1/sso2Δ::hphNT1 [YIP sso1 S79A URA3]/[YIP sso1 S79A URA3]* | This study |
|  | *leu2Δ0/LEU2 lys2Δ0/LYS2 sso1Δ::kanMX4/sso1Δ::kanMX4/ sso2Δ::hphNT1/sso2Δ::hphNT1* |  |
| H3973 | *MAT***a***/MAT*α *ura3::(YIp SSO2 wt URA3)/* *ura3::(YIp SSO2 wt URA3)*  *ura3/ura3 leu2Δ0/LEU2 lys2Δ0/LYS2 sso1Δ::kanMX4/sso1Δ::kanMX4 sso2Δ::hphNT1/sso2Δ::hphNT1 [YIP sso1 S79A URA3]/[YIP sso1 S79A URA3] sso2Δ::hphNT1/sso2Δ::hphNT1 [YIP sso1 S79A URA3]/[YIP sso1 S79A URA3]* | This study |
|  | *leu2Δ0/LEU2 lys2Δ0/LYS2 sso1Δ::kanMX4/sso1Δ::kanMX4 sso2Δ::hphNT1/sso2Δ::hphNT1* |  |
